# Polycystic ovary syndrome and iron overload: biochemical link and underlying mechanisms with potential novel therapeutic avenues

**DOI:** 10.1042/BSR20212234

**Published:** 2023-01-19

**Authors:** Marilyn Mathew, Sathish Sivaprakasam, Jennifer L. Phy, Yangzom D. Bhutia, Vadivel Ganapathy

**Affiliations:** 1 Department of Cell Biology and Biochemistry, Texas Tech University Health Sciences Center, Lubbock, TX, U.S.A.; 2 Department of Obstetrics and Gynecology, Texas Tech University Health Sciences Center, Lubbock, TX, U.S.A.

**Keywords:** bacterial dysbiosis, hemochromatosis, hepcidin, iron overload, polycystic ovary syndrome, probiotics

## Abstract

Polycystic ovary syndrome (PCOS) is an endocrine and metabolic disorder in women with components of significant genetic predisposition and possibly multiple, but not yet clearly defined, triggers. This disorder shares several clinical features with hemochromatosis, a genetically defined inheritable disorder of iron overload, which includes insulin resistance, increased adiposity, diabetes, fatty liver, infertility, and hyperandrogenism. A notable difference between the two disorders, however, is that the clinical symptoms in PCOS appear at much younger age whereas they become evident in hemochromatosis at a much later age. Nonetheless, noticeable accumulation of excess iron in the body is a common finding in both disorders even at adolescence. Hepcidin, the iron-regulatory hormone secreted by the liver, is reduced in both disorders and consequently increases intestinal iron absorption. Recent studies have shown that gut bacteria play a critical role in the control of iron absorption in the intestine. As dysbiosis is a common finding between PCOS and hemochromatosis, changes in bacterial composition in the gut may represent another cause for iron overload in both diseases via increased iron absorption. This raises the possibility that strategies to prevent accumulation of excess iron with iron chelators and/or probiotics may have therapeutic potential in the management of polycystic ovary syndrome.

## PCOS: the disease and clinical features

Polycystic ovary syndrome (PCOS) is a multifaceted disorder that presents as irregular menstruation, high androgen levels, and polycystic appearance of ovaries as detected by ultrasound [1–4]. Anywhere between 3 and 15% of women in reproductive age are affected by this condition worldwide. While the etiology of this disorder remains still elusive, various theories have been postulated as the root cause such as genetics, environmental factors, and metabolic/hormonal disturbances [5–9]. It is very likely that PCOS has a genetic component with involvement of multiple genes; even though the disorder does not have a clearly recognizable hereditary pattern, it does exhibit some form of familial association because a significant fraction of women with PCOS have an affected family member, normally a mother or sister. Sons of mothers with PCOS have also been found to have increased body weight at infancy and insulin resistance later progressing to Type 2 diabetes than those born to mothers without PCOS [10–14]. Clinically, PCOS is currently diagnosed by the following criteria: oligo and/or anovulation, signs of hyperandrogenism, and polycystic ovaries by ultrasound [15]. Oligomenorrhea (abnormal or irregular menstrual periods) and menstrual cycles with no ovulation decrease the reproductive potential in affected women. The long-term effects of PCOS extend beyond the reproductive age because of the higher prevalence of obesity, insulin resistance, diabetes, and cardiovascular diseases accompanied with PCOS [16]. One of theprimary hormonal disturbances in PCOS is hyperandrogenism, which is the underlying cause of the dermatologic abnormalities such as hirsutism (excessive facial and/or body hair), acne, hyperpigmentation, and androgenic alopecia (i.e., male pattern baldness typically associated with thinner and shorter hair, which starts at the hairline and gradually spreads backward) are often seen in women with PCOS [17,18]. The elevated androgen levels are due to overproduction of the hormone in theca cells of the ovary or in the adrenal cortex.

## PCOS: the endocrine disturbances

The endocrine disturbances in PCOS are complex. Insulin resistance causes elevated levels of insulin in circulation. Hyperandrogenism seen in PCOS involves both testosterone as well as dehydroepiandrosterone. Insulin resistance is associated with increased adiposity, which results in increased aromatization of testosterone into estrogen via aromatase in adipocytes. There are also disturbances in the peptide hormones LH (luteinizing hormone) and FSH (follicle-stimulating hormone) that play a pivotal role in ovarian function and ovulation [19,20]. LH and FSH are secreted by the pituitary gland in pulses in response to GnRH (gonadotropin releasing hormone) pulses from the hypothalamus. Interestingly, fast pulses of GnRH favor LH secretion whereas slow pulses favor FSH secretion [21]. LH controls the production of estrogen and progesterone in the ovary while FSH controls follicular development. In women with normal ovarian function, LH and FSH levels are approximately equal during the early part of the menstrual cycle prior to ovulation, but in women with PCOS, LH levels are higher than FSH levels, almost to a 2:1 ratio even when the levels of both hormones are within the normal physiological range [22,23]. This underlines the importance of the LH-to-FSH ratio rather than the absolute levels of each hormone in the dysregulation of ovulation in women with PCOS. In normal woman, a surge in LH secretion precedes ovulation. In contrast, in women with PCOS, the LH levels are higher even prior to ovulation and there is no distinct LH surge or spike, which disrupts ovulation. This happens because GnRH secretion is relatively fast in women with PCOS and it does not slowdown in response to estrogen or progesterone in the negative feedback loop. It is this faster-than-normal release of GnRH in the hypothalamus that promotes increased secretion of LH in relation to FSH, thus leading to an increased LH-to-FSH ratio. Theca cells in the ovary possess the enzymatic machinery to produce androgens in response to LH, and the elevated levels of LH in PCOS constitute the primary cause for hyperandrogenism seen in this disorder.

## PCOS: the connection to iron

Several proteins connected to the biology of iron are altered in blood in patients with PCOS, and most of these changes strongly suggest the presence of mild iron overload in this disease [24]. The changes in four of these proteins are important to understand the connection of PCOS to iron: ferritin, haptoglobin, hepcidin, and α2-macroglobulin.

Ferritin is an iron-storage protein within cells, and the levels of this protein inside and outside the cell rise under conditions of excess iron in the system [25]. Even though iron is an essential element obligatory for cell survival, it is highly oxidative, capable of generating reactive oxygen species. As such, the window for the range of iron concentration in biological systems is narrow for optimal beneficial effects with minimized detrimental consequences. Therefore, when the levels of iron rise as in iron-overload conditions, it has to be stored in an inert form, and this happens in the form of Fe^3+^ complexed with ferritin. In terms of oxidative potential, the divalent Fe^2+^ is active and the trivalent Fe^3+^ is inert. Hemoglobin and myoglobin contain mostly Fe^2+^ in the form of heme, and it is the divalent form in this protein that binds molecular oxygen. Iron in cytochromes in the electron transport chain and in the xenobiotic-metabolizing machinery (cytochrome P450s), iron alternates between the two valency states, Fe^2+^ and Fe^3+^, because of the involvement of these proteins in electron transfer reactions; when an electron leaves the system, Fe^2+^ becomes Fe^3+^ and when an electron is added to the system, Fe^3+^ becomes Fe^2+^. Ferritin stores iron solely in the form of Fe^3+^. Under iron-overload conditions, ferritin levels rise to sequester iron in Fe^3+^ form so that the excess iron will not participate in the generation of reactive oxygen species. Ferritin consists of a heavy chain and a light chain, and the levels of both increase in response to excess iron. This change is made possible due to the presence of a binding site for iron-responsive proteins IRP1/2 in the 5′-UTR of mRNAs for the heavy and light chains of ferritin [26,27]. IRPs function as iron-responsive proteins when not bound to iron whereas they cease to function as iron-responsive proteins when bound to iron. Consequently, the binding of IRPs to the 5′-UTR of ferritin mRNAs is decreased under conditions of excess iron, thus promoting protein translation. As such, elevated levels of ferritin in the plasma of patients with PCOS [24] suggest an increase in iron levels.

Haptoglobin is a protein in the plasma that has high affinity for hemoglobin [28]. When circulating levels of cell-free hemoglobin are elevated in conditions such as hemolytic anemia, it binds to haptoglobin, and the complex is rapidly cleared from blood by liver. Thus, a decrease in plasma levels of haptoglobin indicates more than normal release of free hemoglobin into circulation. Macrophages take up free hemoglobin and degrade the heme moiety with the enzyme heme oxygenase-1 and release iron into circulation. Therefore, decreased levels of haptoglobin in plasma are indicative of excess iron in the system. Deletion of haptoglobin in mice leads to iron overload [29]; since free hemoglobin induces the expression of ferroportin, the iron exporter in the basolateral membrane of the duodenal enterocytes [29], haptoglobin deficiency means increased levels of hemoglobin in circulation and hence increased iron export from the intestine into blood. At least in some studies, the plasma levels of haptoglobin are significantly reduced in patients with PCOS [30,31]. Furthermore, a common polymorphism in the gene coding for the α-chain of haptoglobin is associated with PCOS and this particular polymorphism results in decreased expression of haptoglobin [32]. This iron-regulatory protein not only promotes intestinal iron absorption but also possesses antioxidant and anti-inflammatory properties [32]; therefore, the decreased levels of this protein in circulation in PCOS would be associated with an increase in the intestinal absorption of dietary iron through free hemoglobin and also with increased oxidative stress and inflammation.

Hepcidin is the most important iron-regulatory hormone in the body and it is produced by the liver [33,34]. It is a 24-amino acid peptide that targets the iron exporter ferroportin (also known as SLC40A1 or solute carrier gene family 40, member A1) in cell types such as the enterocytes of the duodenum and macrophages. When bound to hepcidin, the cell-surface ferroportin gets internalized and undergoes degradation. This means that the function of hepcidin is to maintain the levels of iron in the circulation at low levels because it prevents the release of iron from the enterocytes and macrophages into blood. Consequently, the release of hepcidin by the liver and hence the circulating levels of hepcidin are lower than normal when iron levels in the plasma are elevated. Some, but not all, studies have documented that patients with PCOS have reduced levels of hepcidin in the plasma [35,36]. This suggests that the intestinal absorption of dietary iron is promoted and also that macrophages release hemoglobin-derived iron into circulation much more effectively, both processes leading to an increase in iron in the circulation. Iron in circulation is mostly bound to transferrin, with free iron present only in the low micromolar range. Therefore, iron status in the plasma is commonly evaluated by the percent of transferrin saturation with iron; when plasma iron levels are higher than normal, transferrin saturation is increased. All of these parameters supportive of iron excess have been documented in patients with PCOS [37] even though in this particular study circulating levels of hepcidin were increased, rather than decreased, in PCOS patients. The discrepancies in hepcidin levels despite the fact that the presence of excess iron in PCOS is almost unequivocal could be due to the complex relationship between this hormone and iron status. When the defect in hepcidin expression is the first event, it leads to iron excess; in contrast, when the excessive accumulation of iron is the first event, it leads to increase in hepcidin expression. As PCOS is a multifactorial disorder with a wide range of potential etiologic factors, decreased levels of hepcidin in circulation might not be a universal finding, but excess iron is.

Recent studies have shown that hepcidin in circulation is mostly bound to α2-macroglobulin [38]. Interestingly, hepcidin bound to α2-macroglobulin is more effective than free hepcidin in reducing the cell-surface density of ferroportin [39]. In one study, patients with PCOS have reduced levels of α2-macroglobulin in the plasma [31], meaning that the efficacy of hepcidin to bind ferroportin and promote its degradation is compromised in PCOS patients. Thus, the decrease in the circulating levels of hepcidin as well as α2-macroglobulin in patients with PCOS produces a synergistic effect in increasing the density of the iron exporter ferroportin in the cell surface of duodenal enterocytes and macrophages, leading to an increase in circulating levels of iron. However, a later study could not confirm the decrease in circulating levels of α2-macroglobulin in PCOS patients [40], thus underlining the need for additional studies in this area.

Even though most studies have found biochemical parameters in the plasma of patients with PCOS that are indicative of excess iron, the iron overload is only mild. This is reflected in a recent study in which the increase in the levels of iron, hemoglobin, ferritin, and transferrin saturation was in support of excess iron in PCOS, but hepcidin levels were actually increased with no statistically significant change in transferrin iron-binding capacity [37]. Some of these ambiguities might be due to heterogeneity of the disease itself: PCOS patients could present without hyperandrogenemia and insulin resistance, with hyperandrogenemia but without insulin resistance, without hyperandrogenemia but with insulin resistance, and with both hyperandrogenemia and insulin resistance [37]. The severity of menstrual bleeding also needs to be taken into account when interpreting the data on various biochemical parameters in the plasma of patients with PCOS that are relevant to iron homeostasis.

## Hemochromatosis: the genetics and clinical features

Hemochromatosis is an autosomal recessive disorder of iron homeostasis with age-dependent accumulation of excess iron in blood and in tissues [41–43]. It is a single-gene disorder, meaning that mutation in a single specific gene is sufficient to cause the disease, but there are five genes that are independently related to hemochromatosis: HFE, ferroportin, hemojuvelin, transferrin receptor 1, and hepcidin [41–43]. Mutations in the first two genes cause the classical hemochromatosis where excessive iron accumulation takes decades to build up and the clinical consequences of excess iron are not apparent until 50–60 years of age. In contrast, mutations in the latter three genes cause juvenile hemochromatosis where iron accumulation to detrimental levels and the consequent clinical symptoms become evident at a much younger age. Among these five genes, HFE is involved in approximately 85% cases of hemochromatosis. HFE is an iron-regulatory protein and is expressed in a wide variety of tissues. It functions as a gatekeeper for cellular iron uptake by interacting with transferrin receptors on the cell surface. Iron circulates in blood mostly bound to transferrin, and cellular uptake of transferrin-bound iron occurs in most systemic tissues via receptor-mediated endocytosis via the transferrin receptor on the cell surface. HFE interacts with transferrin receptors and keeps the iron entry process under check. Mutations in HFE interfere with this checkpoint and consequently entry of transferrin-bound iron into cells is enhanced with resultant accumulation of iron inside the cells beyond normal levels. The trafficking of HFE to the plasma membrane requires a chaperone protein, which is β2-microglobulin. One of the predominant disease-causing mutations in HFE, C282Y, interferes with the interaction of HFE with β2-microglobulin, thus reducing the cell-surface density of HFE with consequent removal of the regulatory checkpoint on cellular iron entry. The second most prevalent mutation is H63D, which also leads to inactivation of the physiological function of HFE, but the exact mechanism remains unknown.

Hemochromatosis is associated with iron overload not only in most tissues but also in blood. If mutations in HFE promotes iron uptake into tissues to cause excessive iron accumulation inside the cells, one would expect a decrease in iron levels in the circulation. This is however not the case. The molecular basis of excess iron in circulation involves the iron-regulatory hormone hepcidin. Liver is the primary source of hepcidin. It targets the cell-surface transporter ferroportin, which is the only iron exporter known to date in mammalian cells [33,34]. When hepcidin binds ferroportin, the complex gets internalized and degraded. As such, hepcidin prevents the release of iron from the cells. When liver cells take up more iron due to mutant HFE, hepcidin production is decreased, which has consequences in terms of ferroportin function particularly in two cell types: macrophages and duodenal enterocytes. With low levels of hepcidin as occurs in hemochromatosis, ferroportin in these two tissues escape degradation. Macrophages play an obligatory role in iron homeostasis because of their involvement in the recycling of iron in hemoglobin. Two-thirds of the total iron pool in the body is associated with heme in hemoglobin. When erythrocytes are turned over after their normal lifespan (∼120 days), the process takes place in macrophages present in spleen, and the iron released from the degradation of heme is then exported into circulation via ferroportin. In hemochromatosis, the decreased levels of hepcidin increase the function of ferroportin in these macrophages, thus releasing more than normal levels of iron into circulation. This represents one of the two major causes of excess iron in circulation. As a result, intracellular content of iron in macrophages is lower than normal in patients with hemochromatosis. This is in stark contrast with most other systemic organs in which iron gets overloaded in hemochromatosis.

**Figure 1:**
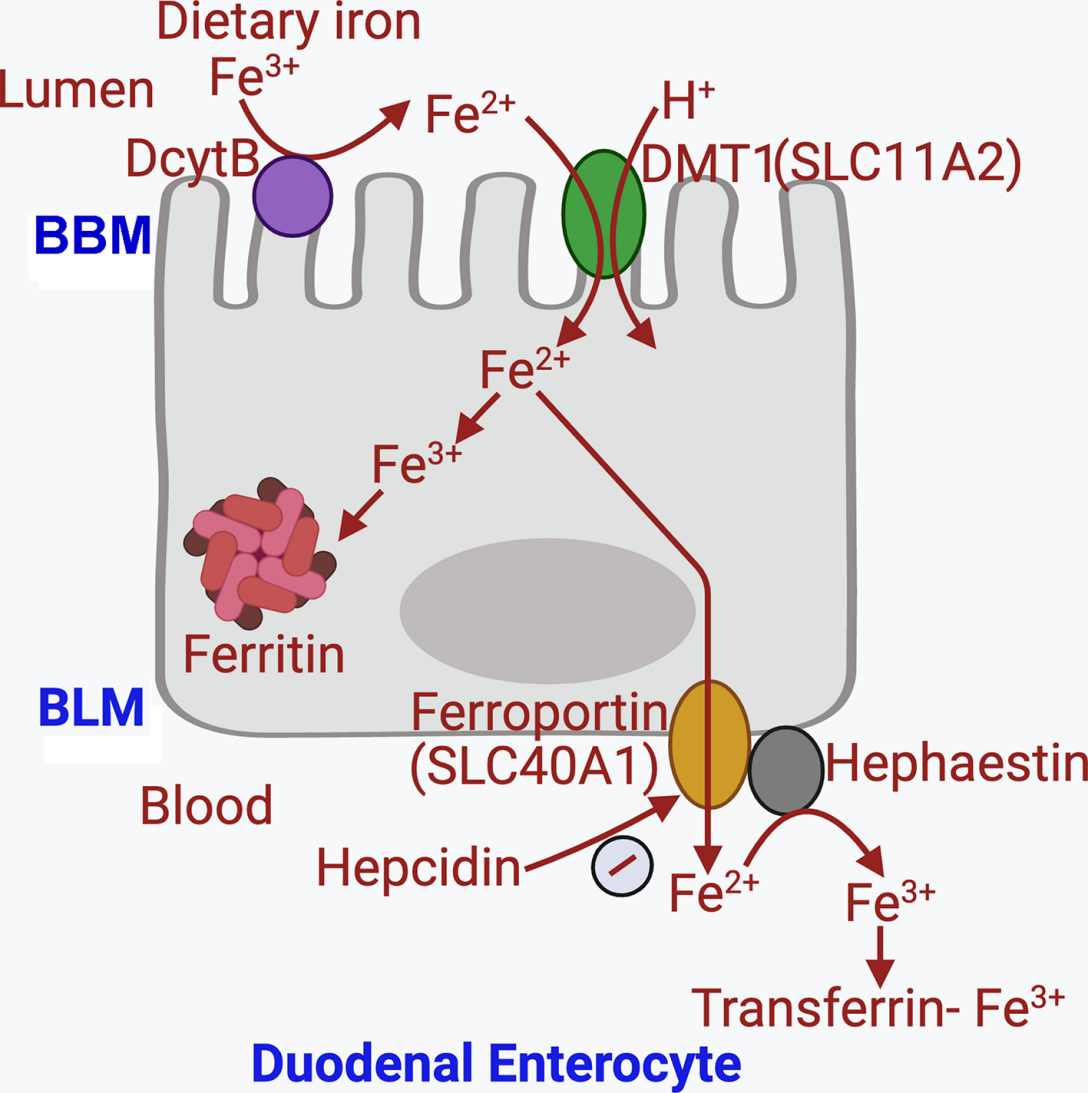
Absorption of dietary iron in the proximal intestine Abbreviations: BBM, brush-border membrane; BLM, basolateral membrane; DcytB, duodenal cytochrome B; DMT1, divalent metal transporter 1 (also called SLC11A2); SLC, solute carrier.

Ferroportin is also responsible for the export of iron from the duodenal enterocytes into blood. This is the terminal step in the intestinal absorption of dietary iron (Figure 1). The absorption of dietary iron occurs predominantly in the duodenum. Iron in the diet exists mostly in its oxidized form, Fe^3+^, which cannot be absorbed. It has to be first reduced to Fe^2+^ before absorption could occur. The lumen-facing apical (brush border) membrane of the absorptive cells (enterocytes) in duodenum expresses an iron-reducing system mediated by DCytB (a cytochrome in the duodenum) that converts Fe^3+^ into Fe^2+^. Vitamin C (ascorbic acid) is also capable of this function. Fe^2+^ is then transported into the cells via the divalent metal ion transporter DMT1 (also known as SLC11A2 – solute carrier family 11, member A2), which is a H^+^-coupled transporter [44]. The microclimate acid pH that normally exists on the luminal surface of the apical membrane [45] provides the necessary inward-directed H^+^ gradient to drive this transporter. Once Fe^2+^ enters the duodenal enterocytes, it is either stored inside the cells or exported out into blood. For storage inside the cells, it has to be first oxidized back to Fe^3+^ which then binds to ferritin. Accordingly, when cellular iron levels go up beyond normal physiological concentrations, ferritin levels also go up. For export out into blood, Fe^2+^ is handled by the iron exporter ferroportin in the blood-facing basolateral membrane. No driving force is involved in this process. This export is always coupled to oxidation of iron from Fe^2+^ to Fe^3+^ on the external surface of the basolateral membrane, catalyzed by hephaestin, which has ferroxidase activity [46]. The oxidized iron (Fe^3+^) then binds to transferrin to circulate in blood. In hemochromatosis, decreased production of hepcidin in the liver leads to enhanced density of ferroportin in duodenum, thus promoting the release of diet-derived iron from the enterocytes into blood. As such, intestinal absorption of iron is accelerated in patients with hemochromatosis, causing iron overload in circulation. As expected, the cellular content of iron in duodenal enterocytes is decreased in hemochromatosis, a situation similar to what is seen with macrophages. When the concentration of transferrin-bound iron is elevated in blood, iron uptake into cells is enhanced, facilitated further by the deficiency of the normal checkpoint function of HFE. Therefore, it becomes obvious that in patients with hemochromatosis, iron levels in circulation increase and iron accumulation in tissues increases. The only two notable exceptions in this general phenomenon are the macrophages and duodenal enterocytes, where iron cellular content actually decreases in hemochromatosis.

As multiple tissues accumulate iron to excessive amounts, clinical symptoms in hemochromatosis are broad, involving several organ functions [41,43,47–50]. The common clinical features include liver cirrhosis, liver cancer, diabetes, cardiomyopathy, nephropathy, arthritis, pituitary dysfunction, sexual dysfunction, and potential association with neurodegenerative diseases such as Alzheimer’s disease and Parkinson’s disease. Using a mouse model of hemochromatosis (*Hfe*-null mice) which phenocopies the human disease, we demonstrated additional pathologic features in the disorder: visual dysfunction [51], colitis and colon cancer [52], gout [53], and diabetic nephropathy [54]. There seems to be some variability in the clinical spectrum depending on whether the disease is caused by C282Y mutation or H63D mutation. Liver disease and infertility are more common with C282Y mutation whereas arthritis, heart disease and neurodegeneration are more common with H63 mutation. As mentioned earlier, the accumulation of iron to detrimental levels in tissues in HFE-associated hemochromatosis takes decades and the clinical symptoms begin to appear only at 50–60 years of age. There is also a significant difference between males and females in disease presentation. The clinical consequences appear relatively earlier in males than in females because of the iron loss in females via menstrual periods during the reproductive age. Other than blood loss, there is no other significant mechanism for elimination of iron from the body. The molecular mechanism of iron toxicity in tissues that accumulate iron excessively is the function of Fe^2+^ as a potent oxidant. This divalent iron is capable of mediating the Fenton reaction that involves the generation of a highly reactive hydroxyl radical in the presence of H_2_O_2_ (Fe^2+^ + H_2_O_2_ → Fe^3+^ + OH^−^ + OH^•^). The hydroxyl radical generates lipid peroxides from membrane-associated polyunsaturated fatty acids, thus causing membrane damage. This process is called lipid peroxidation, which can lead to a form of cell death known as ferroptosis [55,56].

It needs to be noted here that hemochromatosis is very likely the most prevalent genetic disease in Caucasians and Hispanics [57–59]. In Caucasians, the C282Y and H63D mutations are most frequent with the frequency of homozygosity and compound heterozygosity for these two mutations being approximately 1 in 250. In Hispanics, H63D mutation is more common than C282Y mutation with the frequency of homozygosity and compound heterozygosity for these two mutations being approximately 1 in 100.

## PCOS and hemochromatosis: similarities and differences in clinical features

Even though PCOS and hemochromatosis are two distinct disorders, there are striking similarities in the clinical phenotype between the two (Table 1). This includes Type 2 diabetes, insulin resistance, non-alcoholic fatty liver disease, increased risk for breast cancer, pituitary dysfunction, infertility, increased body weight gain, abnormal skin pigmentation, and iron overload. It is tempting to speculate that increased body weight, which is common between PCOS and hemochromatosis, might explain most of the other common clinical features in the two disorders. Expansion of adiposity might lead to increased extragonadal production of estrogen via adipocyte-associated aromatase, which could explain increased risk for breast cancer and differential release of LH and FSH by the pituitary. While estrogen and testosterone suppress the release of LH, the release of FSH is suppressed only by estrogen [60,61]. This differential negative-feedback regulation of the two gonadotrophs by estrogens and androgens might underlie the increased LH:FSH ratio in PCOS and most likely also in hemochromatosis despite the fact that the levels of both LH and FSH are within the normal limits. Presence of excess iron in circulation also seems to be a common feature between PCOS and hemochromatosis even though the magnitude of iron accumulation is much milder in PCOS than in hemochromatosis. While the circulating levels of hepcidin are unequivocally decreased in hemochromatosis, there seems to no consistency in published data on hepcidin levels in PCOS, with some studies showing decreased levels while others showing either no change or an increase. As mentioned previously, hemochromatosis is a single-gene disorder with a defined genotype, which could explain the consistency in data related to hepcidin levels in this disorder. In contrast, PCOS is a heterogenous disorder with potential involvement of multiple factors, both genetic and non-genetic. The dysregulated menstrual cycle in PCOS subjects adds another layer of confounding factors on iron status in circulation and on hepcidin levels. Furthermore, the presence or absence of excess adiposity in PCOS might impact liver function with varied accumulation of fat in liver cells, which might also alter hepcidin production.

**Table 1:** Similarities and differences in clinical features between PCOS and hemochromatosis

Clinical feature	PCOS	Hemochromatosis
Insulin resistance	✓	✓
Diabetes	✓	✓
Pituitary dysfunction	✓	✓
Infertility	✓	✓
Iron overload	✓	✓
Weight gain	✓	✓
Fatty liver	✓	✓
Breast cancer risk	✓	✓
Skin pigmentation	✓	✓
Cysts in ovaries	✓	
Endometrial cancer risk	✓	
Liver cirrhosis		✓
Arthralgia		✓
Cardiomyopathy		✓
Nephropathy		✓

Despite the marked similarities in a number of clinical features between PCOS and hemochromatosis, notable differences exist between the two disorders (Table 1). Arthritis and cardiomyopathy are common features in patients with hemochromatosis but not in patients with PCOS. This is most likely due to the differences in the severity of iron overload in hemochromatosis versus PCOS. The mild increase in iron accumulation in PCOS may not be sufficient to cause damage to joints and cardiac tissue. In contrast, the chronic accumulation of excess iron as occurs in hemochromatosis results in toxic levels of iron at these tissue sites, thus compromising their normal function. PCOS also exhibits certain clinical features that are not common in hemochromatosis. Appearance of structural abnormalities in the ovary and increased risk of endometrial cancer are more common in PCOS than in hemochromatosis; however, it is difficult to pinpoint the underlying mechanism for this difference.

PCOS is common in women with hemochromatosis caused by homozygosity of the C282Y mutation [62]. However, there appears to be no significant increase in the prevalence of HFE mutations in PCOS patients compared with that in general population [63]. Again, this seemingly discrepant findings are expected given the fact that PCOS is not a single-gene disorder with etiologic contributions from genetic and non-genetic factors.

## PCOS and hemochromatosis: similar therapeutic efficacy of phlebotomy

Lifestyle changes such as appropriate diet and exercise to reduce body weight are the first step for obese patients with PCOS. Pharmacologic intervention includes combined estrogen-progestin oral contraceptives for managing hyperandrogenemia and menstrual dysfunction. Metformin, the widely used insulin sensitizer, is an alternative to oral contraceptives to correct disturbances in menstrual cycle. Metformin as well as thiazolidinediones, both of which are insulin sensitizers, improve insulin resistance in PCOS. For women with PCOS who aspire to become pregnant, clomiphene citrate or letrozole are used to induce ovulation [64,65]. Clomiphene is a non-steroidal Selective Estrogen Receptor Modulator (SERM) that antagonizes the action of estrogen on the hypothalamus–pituitary axis. Letrozole on the other hand is a potent inhibitor of aromatase, thus reducing estrogen production. FSH promotes egg maturation and ovulation. Recent clinical trials have shown that phlebotomy is effective in restoring certain specific parameters in women with PCOS [66]. The present study also compared the effects of phlebotomy versus oral contraceptives. Both treatments showed similar efficacy in reversing insulin resistance and hyperandrogenemia. However, there were some differences. Phlebotomy showed less changes in lipid profile in plasma whereas oral contraceptives were more effective than phlebotomy in treating disturbances in menstrual cycle. However, the beneficial effects of phlebotomy with resultant decrease in body iron stores were not seen in women with PCOS on oral contraceptives [67,68]. In patients with hemochromatosis, periodic blood letting (i.e., phlebotomy) is the mainstay of treatment. Since iron overload appears to be a common feature in PCOS and hemochromatosis, it is not surprising that phlebotomy does have beneficial effects in both disorders, though variable in PCOS depending on the other concurrent drug treatments.

## Impact of chronic exposure to excess iron on ovary and folliculogenesis

**Figure 2:**
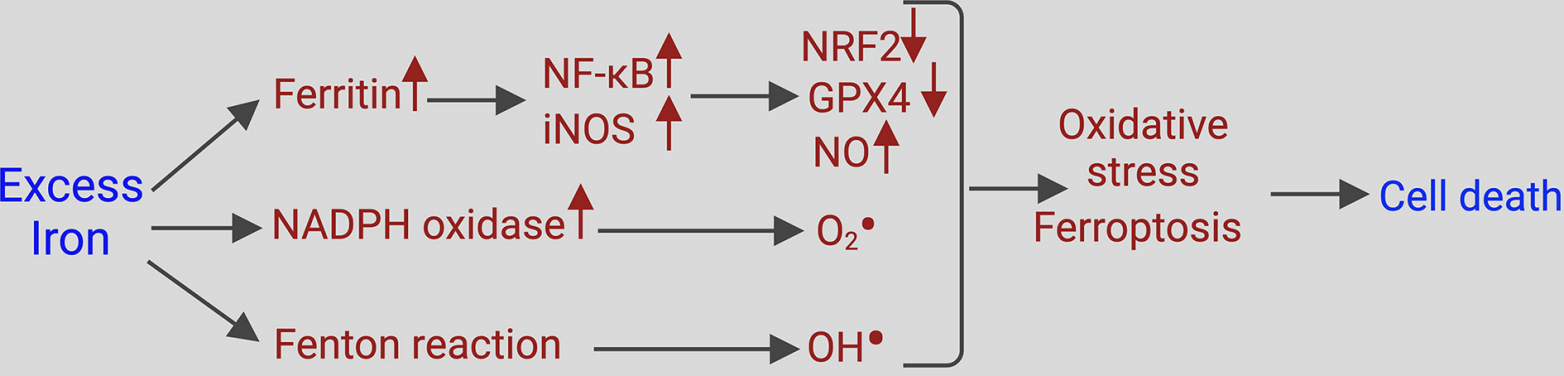
Molecular mechanisms underlying the defective granulosa cell function and cell death induced by exposure to excess iron Abbreviations: GPX4, glutathione peroxidase 4; iNOS, inducible nitric oxide synthase; NF-κB, nuclear factor κB; NRF2, nuclear factor erythroid 2-related factor 2.

There are a few reports in the literature that provide evidence for detrimental effects of excess iron on ovarial granulosa cells and consequently on folliculogenesis [69–71]. Exposure of granulosa cells to follicular fluid containing excess iron induces cell death via iron-initiated ferroptosis [69]. This detrimental effect is indeed mediated by iron-induced oxidative stress because antioxidants such as vitamin E or iron chelators prevent this effect. Interestingly, the iron-induced oxidative stress involves not only the Fenton reaction but also the activation of the plasma membrane enzyme complex NADPH oxidase, which generates superoxide by transferring electrons to molecular oxygen [70]. There is also mitophagy associated with degradation of the antioxidant enzyme glutathione peroxidase 4 [70]. Furthermore, iron overload in granulosa cells up-regulates the proinflammatory transcription factor NF-κB and, as a result, inducible nitric oxide synthase [71]. The resultant increase in nitric oxide suppresses estrogen production and thus interferes with normal folliculogenesis and ovum maturation. This effect of nitric oxide on estrogen production by granulosa cells is due to suppression of expression and activity of the enzyme aromatase that converts androgens into estrogens [72,73]. The suppression of aromatase expression appears to be due to the ability of nitric oxide to increase cGMP and decrease cAMP [72] whereas the inhibition of the catalytic activity of the enzyme seems to involve covalent modification of a critical cysteine-associated thiol group with nitric oxide [73]. These reports provide evidence and plausible mechanisms for the detrimental effects of excess iron on ovarian function (Figure 2). The decrease in aromatase expression and activity might also explain the excess production of androgens when ovaries are exposed to excess iron.

## Dysbiosis in PCOS and hemochromatosis: a possible connection to aberrant intestinal iron absorption

**Figure 3:**
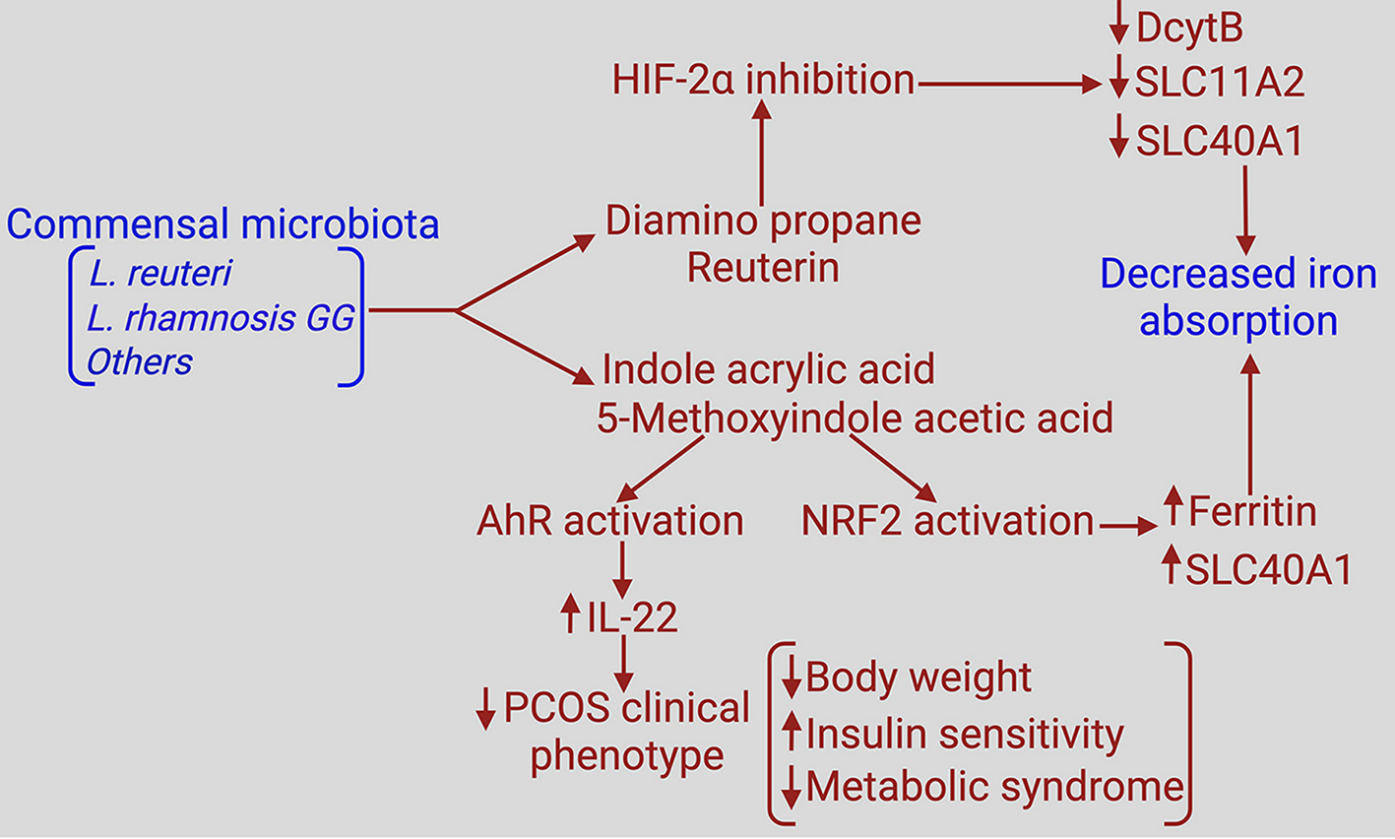
Modulation of intestinal iron absorption and PCOS phenotype by colonic bacteria and their metabolites Abbreviations: AhR, aryl hydrocarbon receptor; DcytB, duodenal cytochrome B; HIF-1α, hypoxia-inducible factor-1α; NRF2, nuclear factor erythroid 2-related factor 2; PCOS, polycystic ovary syndrome; SLC, solute carrier.

The capacity of the small intestine to absorb dietary iron is limited under physiologic conditions. It varies with the systemic iron status; when systemic iron levels are low, intestinal absorption is accelerated whereas when the systemic levels of iron are high, intestinal absorption is suppressed. Diet contains iron in two forms: heme and free iron. Heme iron is absorbed much more effectively than free iron (15–30% versus 2–20%, respectively) [74]. The mechanism of absorption for heme iron is different from that of free iron. As described in Figure 1, absorption of free iron involves multiple proteins and transporters [75,76]. In hemochromatosis, the decreased level of hepcidin in circulation is primarily responsible for abnormally increased iron absorption in the intestine. In contrast, even though there is evidence of excess iron in PCOS, it is not clear if changes in hepcidin are responsible for any altered iron absorption in the intestine. As mentioned above, the decreased hepcidin is not a universal finding in PCOS. This brings the potential role of colonic bacteria as a regulator of intestinal iron absorption. Recent studies have shown that normal indigenous bacteria in colon produce specific metabolites that appear in systemic blood and inhibit iron absorption in the small intestine [77]. Two of these metabolites are diaminopropane and reuterin, both of which are inhibitors of hypoxia inducible factor-2 and as a result decrease the expression of DMT1 (SLC11A2), duodenal ferrireductase DCytB, and the iron exporter ferroportin (SLC40A1) and increase the expression of the iron-storage protein ferritin (Figure 3). The end result of these changes is decreased delivery of dietary iron from the lumen into blood. Two other bacterial metabolites, namely indole acrylic acid and 5-methoxyindole acetic acid, are activators of the anti-oxidant transcription factor NRF2 [78–80], which also plays a pivotal role in the regulation of iron absorption in the intestine. Ferroportin and ferritin are the transcriptional targets for NRF2 [81]. Induction of ferritin in the duodenal enterocytes by these bacterial metabolites decrease iron delivery into blood whereas induction of ferroportin enhances iron delivery into blood. Even though these two events seem on the surface contradictory, it is understandable given that the primary physiological function of NRF2 is to block oxidative stress within the cell. Free iron is a pro-oxidant, and NRF2 blocks this iron-dependent oxidative stress by two mechanisms, first by increasing the sequestration of free iron in ferritin and second by promoting its removal from the cells via ferroportin. On the whole, it seems logical to conclude that the net result of the four metabolites from colonic bacteria is a decrease in intestinal iron absorption. Two specific bacterial strains produce diaminopropane, reuterin, indole acrylic acid, and 5-methoxyindole acetic acid [78–80]; these are *Lactobacillus reuteri* and *Lactobacillus rhamnosus GG*. There may be additional bacterial strains in the colon that are capable of generating the same metabolites. Bacterial dysbiosis occurs both in PCOS [82–86] and in hemochromatosis [52]. This is associated with decreased abundance of *Lactobacilli* in PCOS [85]. Despite the demonstration of dysbiosis in a hemochromatosis mouse model [52], there is no information available if *Lactobacilli* abundance is specifically decreased. A decrease in this bacterial strain is expected to decrease the circulating levels of diaminopropane, reuterin, indole acrylic acid and 5-methoxyindole acetic acid in PCOS patients, which would result in attenuation of the inhibitory effects of these metabolites on intestinal iron absorption. Thus, the dysbiosis seen in PCOS might offer a plausible mechanism for increased absorption of dietary iron in the intestine, thus leading to iron overload. *Lactobacilli* also correlate positively with estrogen levels in the host and fecal transplantation of microbiota from normal animals into animals with PCOS phenotype reduces androgen levels [86].

## Relevance of bacterial metabolites to the biological features of PCOS

Indole acrylic acid and 5-methoxyindole acetic acid are bacterial metabolites arising from tryptophan metabolism. These metabolites also function as agonists for another nuclear receptor, known as AhR (aryl hydrocarbon receptor). In the gut, activation of AhR in resident immune cells in the lamina propria by these bacterial metabolites induce the production of the cytokine IL-22 [79]. Gut microbiota and dietary tryptophan have a major influence on IL-22 production in the gut [79]. Recent studies have shown that this cytokine has an important role in the pathogenesis of PCOS [87,88]. Dysbiosis associated with PCOS results in reduced production of IL-22 [87] and exogenous administration of this cytokine was able to reverse many of the clinical phenotypes typical of PCOS such as insulin resistance, abnormal ovarian morphology, and defects in estrous cycle in animal models [88].

Short-chain fatty acids such as acetate, propionate, and butyrate represent another class of important bacterial metabolites that elicit beneficial effects on the host via multiple molecular targets including histone deacetylases and selective G-protein-coupled receptors [89–92]. The metabolites are the end products of bacterial fermentation of dietary fiber. *Bifidobacteria* are particularly capable of generating short-chain fatty acids. Patients with PCOS show lower concentrations of short-chain fatty acids in fecal samples [93]. Cell-surface G-protein-coupled receptors for these metabolites are expressed on the hormone-producing enteroendocrine cells in the gut that secrete hormones such as GLP-1 and peptide YY [94], which improve insulin resistance by acting on pancreatic β cells to promote insulin secretion. The same peptides could also reduce food intake by acting via the gut-brain axis, thus reducing weight gain. As such, short-chain fatty acids generated in the colon via bacterial metabolism have the ability to attenuate at least some of the clinical and biochemical phenotypes associated with PCOS. The lower levels of these metabolites in PCOS patients might play a pathological role in the development of insulin resistance and obesity and other associated clinical features.

## Dietary intervention for the management of PCOS

The above-described microbiome and metabolite studies are consistent with findings that dietary modification such as elimination of starches (primarily dairy, grains, and processed sugars) have favorable effects on body weight, insulin secretion, plasma lipids, and ovulation in women with PCOS [95,96]. Concurrently, it has been found that specific parameters can be modulated by subtle differences in diets to prioritize weight loss by practicing a monounsaturated fat-enriched diet, menstrual regularity by adhering to a low glycemic diet, and improved depression and self-esteem in a diet high in protein [97]. The use of probiotic supplementation was also found to decrease total testosterone and increased sex hormone binding globulin but showed insignificant effects on other metabolic metrics [98]. Given the fact that some of the bacterial metabolites generated by *Lactobacilli* in the colon elicit beneficial effects in PCOS patients in terms of iron status and cytokine secretion are derived from tryptophan, it is tempting to speculate that dietary supplementation of this particular amino acid might have therapeutic efficacy in PCOS. Animal studies have in fact demonstrated that oral administration of tryptophan expands the incidence of *Lactobacilli* in the intestinal tract and increases the secretion of the cytokine IL-22 [79], thus providing supporting evidence for the speculation.

## Conclusion

There is convincing evidence for significant similarities in clinical symptoms between women with PCOS and women with the genetic disorder hemochromatosis. Accumulation of excess iron is the most fundamental common biochemical feature between the two disorders. Obesity, insulin resistance, diabetes, infertility, dysbiosis of colonic bacteria, and decreased *Lactobacilli* species in colon are some of the readily noticeable common findings between PCOS and hemochromatosis. Even though the etiology of PCOS is multifactorial whereas hemochromatosis is a single-gene disorder, it is surprising to see so much commonality in the clinical presentation of the two disorders. Irrespective of the differences in the etiology, dysregulation of intestinal iron absorption, and dysbiosis of colonic microbiome may underlie the pathogenesis in both cases. This highlights phlebotomy, dietary intervention, and probiotics as potential therapeutic avenues for PCOS and hemochromatosis.

## Open Access

Open access for this article was enabled by the participation of Texas Tech University Health Sciences Center in an all-inclusive *Read & Publish* agreement with Portland Press and the Biochemical Society.

## Abbreviations

BBM: brush-border membrane
BLM: basolateral membrane
FSH: follicle-stimulating hormone
HIF-1α: hypoxia-inducible factor-1α
LH: luteinizing hormone
NRF2: nuclear factor erythroid 2-related factor 2
PCOS: polycystic ovary syndrome
SERM: Selective Estrogen Receptor Modulator
SLC: solute carrier
